# Examination of Potential Industry Conflicts of Interest and Disclosures by Contributors to Online Medical Resource Databases

**DOI:** 10.1001/jamanetworkopen.2022.20155

**Published:** 2022-07-05

**Authors:** SooYoung H. VanDeMark, Mia R. Woloszyn, Laura A. Christman, Michael H. Gatusky, Warren S. Lam, Stephanie S. Tilberry, Brian J. Piper

**Affiliations:** 1 Department of Medical Education, Geisinger Commonwealth School of Medicine, Scranton, Pennsylvania; 2 Center for Pharmacy Innovation and Outcomes, Geisinger Health, Forty Fort, Pennsylvania

## Abstract

**Importance:**

Physicians contribute content to online databases, and other health care professionals use these websites to support their decision-making. Financial conflicts of interest (COI) have the potential to adversely impact evidence-based patient care.

**Objectives:**

To quantify the potential COI among content contributors to 2 popular point-of-care medical resources, UpToDate and DynaMed, overall and by gender, and to compare self-reported and industry-mandated disclosures.

**Design, Setting, and Participants:**

This cross-sectional study compiled an initial list of contributors for each website using the Centers for Disease Control and Prevention’s Leading Causes of Death. The top 50 causes were used to identify relevant articles from each database from November 30 to December 7, 2020. The authors and editors of those articles were investigated. Data were analyzed from January 2021 to March 2022.

**Main Outcomes and Measures:**

Self-reported contributor disclosure status was compared with financial remuneration as reported in the Centers for Medicare & Medicaid Services Open Payments (OP) database from 2013 to 2018.

**Results:**

A total of 179 point-of-care database authors and editors were assessed. Combined, they received $77.7 million, with a mean of $583 218 (95% CI, $0-$4 679 651) and median (range) of $29 073 ($10-$17 517 315) each. Most of the compensation ($68.1 million [87.6%]) went to UpToDate contributors. Of 128 UpToDate contributors, 76 (59.4%) reported nothing to disclose, and among these, 44 contributors (57.9%) had a record of receiving a financial payment on OP. Women UpToDate contributors received 2.5% of the total compensation paid from industry. The top 10 UpToDate contributors who received the most financial remuneration earned $56.1 million combined, were all men, and only 1 had a nothing-to-disclose status. Of 51 DynaMed contributors, 42 (82.4%) reported nothing to disclose, and among these, 35 contributors (83.3%) had an OP entry (mean, $79 820; 95% CI, $0-$400 774; median [range], $1403 [$26-$630 424]). Among the top 10 DynaMed contributors, 8 (80.0%) were men. Six of the top 10 DynaMed contributors reported nothing to disclose yet had an OP entry.

**Conclusions and Relevance:**

This cross-sectional study found that contributors to point-of-care databases were the recipients of nearly $78 million from pharmaceutical companies and medical device manufacturers, and these payments were often not disclosed in association with contributed content. Although these findings do not necessarily suggest ethical lapses among the physicians studied, point-of-care resource websites, like UpToDate and DynaMed, should consider implementing more stringent COI policies and employ an unbiased team to verify self-reported disclosure statuses among content contributors against OP reports.

## Key Points

**Question:**

What are the potential conflicts of interest (COI) and concordance levels between self-reported and industry-mandated COI reporting for point-of-care databases?

**Findings:**

In this cross-sectional study including 179 US physicians contributing content to UpToDate and DynaMed, contributors received an appreciable amount of industry support ($77.7 million), with 6 of the top 10 DynaMed contributors reporting nothing to disclose. The top 10 most highly compensated UpToDate contributors were all men.

**Meaning:**

These findings suggest that policies for COI disclosure in point-of-care databases may need strengthening and increased transparency.

## Teaser

This cross-sectional study assesses self-reported and industry-mandated conflict of interest disclosures among contributors to online medical resource databases overall and by gender.

## Introduction

Conflicts of interest (COI) were defined by the Institute of Medicine’s Committee on Conflict of Interest in Medical Research, Education, and Practice as a set of “circumstances that create a risk that professional judgments or actions regarding a primary interest will be unduly influenced by a secondary interest.”^1^ Prior quantitative bioethics research has characterized COI among physician authors of biomedical^2^ and pharmacology textbooks,^3^ psychiatry’s *Diagnostic and Statistical Manual of Mental Disorders* (Fifth Edition),^4^ and clinical practice guidelines.^5,6^ However, there is limited research on the potential COI among authors and editors of online clinical resources, such as UpToDate and DynaMed.^7,8,9^

UpToDate and DynaMed are online, subscription-based products used by physicians to assist in clinical decision-making. Both UpToDate and DynaMed promote their websites as evidence-based resources for physicians to improve patient health outcomes at the point of care.^10,11^ The content on these websites is written, edited, and overseen by various health care professionals. UpToDate and DynaMed each maintain publicly available COI policies that require collecting and reviewing information on any relevant financial relationships of all contributors and their spouses or partners annually.^12,13^ If this information is not provided by the contributor, both databases exclude the contributor from participating. If a financial relationship is disclosed, each database has an editorial team and process that work to resolve any issues. This study focused on characterizing potential COI that could occur when a physician writes or edits medical content (the primary interest) yet could be influenced by financial gain (the secondary interest) in the form of payments made by health care manufacturers to the author.

In an effort to improve transparency and as part of the Physician Payments Sunshine Act, the Centers for Medicare & Medicaid Services (CMS) discloses financial payments from drug, device, biological, and medical supply manufacturers to US-based physicians through its online and publicly accessible database, Open Payments (OP).^14^ These financial transactions are submitted by reporting entities to OP annually, and physicians are encouraged to review their public information regularly, as well as to undergo the process to dispute and correct any of their information.^15^ Another online, publicly accessible database is Dollars for Docs (DFD), which is maintained by the nonprofit, investigative journalism organization, ProPublica. DFD also allows users to look up payments made from relevant manufacturers to US-based physicians as reported to CMS.^16^ OP and DFD report all payments in amounts greater than $10.

A systematic review by Taheri et al^6^ determined that physician self-reporting of COI was highly discrepant with OP and DFD information, but that it was currently unclear if men or women were more likely to have discrepant reports. Examination of the 31 contributors to a nonsubscription-based (ie, free with registration) online point-of-care database for potential COI identified 19 discordant authors (61.3%) who self-reported nothing to disclose but had an OP entry.^17^ Less than one-fifth (18.1%) of authors who contributed entries on the top 50 causes of death were women,^17^ which is congruent with gender disparities among biomedical textbook authors.^2,3^ A small report by Amber et al^7^ focused on UpToDate and DynaMed authors and editors that reviewed 6 articles on each site found no COI among DynaMed contributors, but all UpToDate contributors had COI. Furthermore, UpToDate articles listed the brand names for 17 drugs.^7^ Examination of 23 UpToDate articles for the treatment of Crohn disease and ulcerative colitis determined that almost half of contributors (48%) did not fully disclose their COI.^8^

We investigated potential COI among a broad sample of content contributors for DynaMed and UpToDate by cross-checking their self-reported disclosure against financial records available from OP and verifying these with DFD. Additionally, a descriptive analysis of UpToDate and DynaMed content contributors’ disclosure status, financial compensation, and gender was performed, with further evaluation of each database’s top 10 physician-contributors who received payments.

## Methods

This cross-sectional study was deemed exempt from review and informed consent by the Geisinger institutional review board because it is not considered human participants research. We followed the Strengthening the Reporting of Observational Studies in Epidemiology (STROBE) reporting guideline for observational studies.

### Procedures

UpToDate^18^ and DynaMed were selected based on their favorable ratings in empirical evaluations of the breadth of coverage, timeliness of updating, use of evidence-based methods, and utility;^19,20,21^ because our library subscribed to them; and to extend prior research that showed that COI were prevalent^7,8,9^ with some concerns about disclosure accuracy.^9^ UpToDate has entries for more than 12 000 clinical topics and more than 6900 drug entries, with more than 2 million users in 191 countries and territories.^18^ Using the US Centers for Disease Control and Prevention’s top 50 causes of mortality,^22^ each cause was searched on UpToDate and DynaMed. The research team, with individuals assigned to specific diseases, selected comparable articles from the first page of search results on each site. Only 42 causes of death were used for data collection, resulting in a total of 84 articles reviewed. Causes of death were excluded if they provided no relevant search result on either database (eg, “operations of war and their sequelae”).

Content contributors were defined as the individuals listed on a given UpToDate or DynaMed article page—specific titles for contributors depend on the database, but include: *Author*, *Deputy Editor*, *Section Editor*, *Recommendations Editor*, and *American College of Physicians (ACP) Reviewer*. Each article’s listed content contributors, regardless of title, composed our initial list of contributors. Contributors who were of unknown or international origin were excluded, since OP and DFD only reported on US-based physicians in 2020. Contributors stated nothing to disclose or disclosed the companies from which they have received payment under each article on both resources. This status was collected. All articles and contributor names were compiled between November 30 and December 7, 2020.

Each unique contributor was then searched in OP and DFD. If an entry was found, the financial information for 2013 to 2018 was recorded in accordance with each website’s categorization method. For example, money reported to OP was categorized as *general payments*, *research*, *associated research*, and *owner/investment*. The categories as described by OP are as follows: *general payments* are unassociated with any research studies (eg, speaker and consulting fees, gifts, food and beverage^16^); *research* is for when the physician is the primary recipient and comprises basic and applied research, as well as product development; *associated research* refers to funding paid to a research institute or entity where the physician is named as the principal investigator; and *owner/investment* suggests ownership or investment in a company and includes financial relationships, such as stock options, bonds, and partnership shares.^14^ Contributor gender was verified or cross-checked and documented using the National Provider Identifier registry.^23^

The research team performed a check on the data collected for 20 contributors (11.2%) selected at random to ensure accuracy in the amounts received according to the database, category, and year, as well as the contributor’s gender. No physician contributed content to both UpToDate and DynaMed. Although some contributors were listed on multiple articles, no variability in disclosure status was found between articles. Contributors were classified as discordant if they reported nothing to disclose but were found to have an OP or DFD entry. Conversely, contributors were defined as concordant if they made a disclosure and had an OP or DFD entry.

### Statistical Analysis

Fisher exact tests were performed to determine whether there was an association between a physician-contributor’s gender and disclosure status (for each database). Welch unpaired *t* tests were performed to compare the means between discordant and all contributors per point-of-care databases in each of the 4 OP categories. A Pearson correlation test was performed to assess the correspondence between the data collected for OP and DFD. The monetary data collected for each contributor from OP and DFD were highly correlated; thus, only the findings from OP are described in the results. Data were assessed using Prism version 9.1.0 (GraphPad). A 2-sided *P* < .05 for each test was considered statistically significant. Data were analyzed from January 2021 to March 2022.

## Results

**Figure 1.**
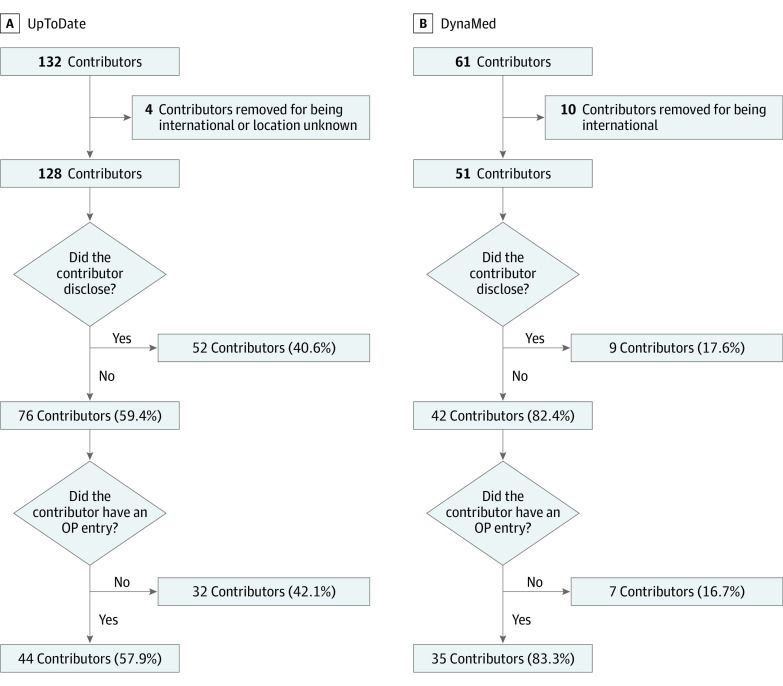
Flowchart of UpToDate and DynaMed Content Contributors, Showing Disclosure Status and the Center for Medicare & Medicaid Services Open Payments (OP) Entry Status

Of 179 US-based physician content contributors (27.9% women) associated with the 84 articles, 128 were from UpToDate and 51 were from DynaMed (Figure 1). Combined, they received $77.7 million, with a mean of $583 218 (95% CI, $0-$4 679 651) and median (range) of $29 073 ($10-$17 517 315) each. Women contributors to UpToDate were less likely to have an OP entry (13 of 34 women [38.2%]) than men (78 of 94 men [83.0%]; χ^2^_1_ = 24.32; *P* < .001), but there was no gender difference among DynaMed contributors. Women UpToDate contributors received 2.5% the total remuneration awarded from industry, women DynaMed authors received 17.3%.

The reported financial payments from industry to OP for the UpToDate contributors totaled to $68 085 233; of which, the top 10 contributors accounted for $56 083 923 (82.4%). The total reported to OP for the DynaMed contributors was $9 576 109, of which the top 10 physician-contributors who received payments accounted for $8 882 249 (92.8%).

Most contributors to UpToDate (76 contributors [59.4%]) and DynaMed (42 contributors [82.4%]) did not disclose any COI related to their article topic (Figure 1). However, of those UpToDate contributors who did not disclose a COI, 44 (57.9%) had an OP entry. This discordance—self-reporting nothing to disclose yet having an OP entry—among UpToDate contributors accounted for a combined $4 811 763, with a mean of $109 358 (95% CI, $0-$1 033 277) and a median (range) of $3608 ($10-$3 120 614) each in payment from the health care industry, or 7.1% of the total compensation (eTable 1 in the Supplement). Similarly, 35 DynaMed contributors (83.3%) who reported nothing to disclose had an OP entry. These discordant DynaMed contributors accounted for a total of $2 793 708, with a mean of $79 820 (95% CI, $0-$400 775) and a median (range) of $1403 ($26-$630 424) each or 29.2% of DynaMed’s total (eTable 2 in the Supplement).

Among UpToDate content contributors, 46 (35.9%) were found to be concordant. Additionally, 6 content contributors (4.7%) were found to be neither discordant nor concordant. They had made a disclosure, but no OP nor DFD record was found. Each of the 9 DynaMed content contributors who disclosed was found to be concordant.

An unpaired Welch *t* test was performed to compare the amount of general payment remuneration received by 44 discordant UpToDate contributors (mean [SD], 80.5% [35.8%] of all renumeration received) against all 90 UpToDate contributors with OP entries (mean [SD], 64.7% [39.2%] of all renumeration received) and found that discordant UpToDate contributors received more remuneration in the form of general payments than all UpToDate authors with OP entries (difference, 15.8%; 95% CI, 2.4%-29.4%; *t*_93_ = 2.3; *P* = .03; ). Another unpaired *t* test found that the amount of associated research payments for the 44 discordant UpToDate authors (mean [SD], 17.1% [34.1%] of all renumeration received) was less than that of all 44 discordant UpToDate authors with OP entries who received remuneration in this category (mean [SD], 30.8% [38.4%] of all renumeration received) (difference, −13.8%; 95% CI, −26.7% to −0.8%; *t*_95_ = 2.1; *P* = .04). No significant association was found between the means in the categories of research payments and ownership/investment for UpToDate. Additional *t* tests found no association between all DynaMed authors and all DynaMed discordant authors in any of the 4 OP financial categories.

**Figure 2.**
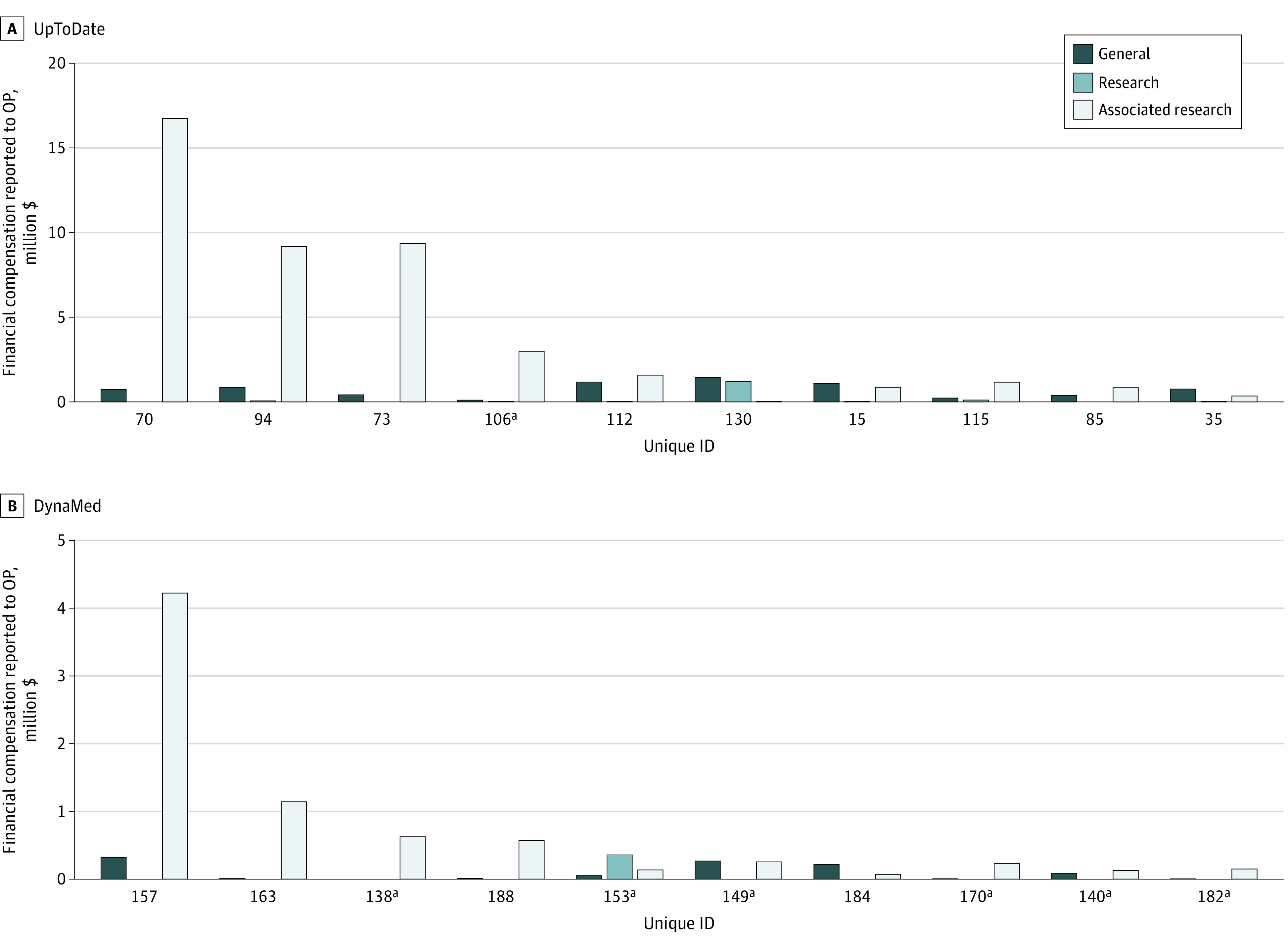
Financial Remuneration by Category as Reported to the Center for Medicare & Medicaid Services Open Payments (OP) for the Top 10 UpToDate and DynaMed Physician-Contributors Who Received Payments Contributors are listed by unique ID. ^a^Indicates the contributor reported nothing to disclose.

All the top 10 physician-contributors who received payments in UpToDate were men, of whom only 1 contributor was discordant. Among the top 10 DynaMed physician-contributors who received payments, 8 were men, and 6 contributors were discordant (Figure 2). Figure 2 also shows a breakdown of the reported compensation in millions to the top 10 physician-contributors of each database who received payments in the 4 OP categories, with associated research dominating payments. Further investigation of the 7 discordant top 10 physician-contributors who received payments found 1 contributor who received payments from a manufacturer for drugs and/or medical devices that was specifically mentioned by brand name in the article to which the contributor was assigned. The financial remuneration to this contributor was $4695.

Among all UpToDate content contributors, 94 (73.4%) were men and 34 (26.6%) were women. A Fischer exact test found that men UpToDate contributors were more likely to report a disclosure than women (45 men [47.9%] vs 7 women [20.6%]). Among DynaMed contributors, 34 (66.7%) were men and 17 (33.3%) were women. There was no difference in how the genders (disclosed among DynaMed contributors (6 men [17.7%] vs 3 women [17.7%]). There was no notable difference in discordance (yes vs no) based on the contributor’s gender for either database.

## Discussion

This cross-sectional study identified appreciable payments from industry (US$77.7 million) to point-of-care contributors with potential room for improvement in self-reported disclosures. In contrast to a more focused prior report,^7^ this study found potential COI among contributors for both point-of-care online resources. This is likely owing to a difference in methods, including sample characteristics. Our findings of discordance in UpToDate (57.9%) and DynaMed (83.3%) are similar to a prior finding of 61.3% discordance among Medscape contributors.^17^ Such high discordance rates and this apparent disagreement between self-reported and mandated reporting suggest a need for further research to fully illuminate this issue, as well as follow-up remediation by these online point-of-care resources. Based on the discordance and the large sums of remuneration among discordant contributors (>$7.5 million) in this study, there is a strong likelihood for there to be potential COI among physician content contributors who self-report, particularly in the category of general payments. Investigation into a subgroup of our sample contributors found evidence suggestive of potential COI. However, it is important to emphasize that the objective of this research was not to ascertain any specific contributor’s COI. A Canadian team of gastroenterologists expressed frustration that UpToDate did not provide sufficient transparency or clarity regarding their COI policies.^9^ If the point-of-care resources provided temporal information about when disclosures were made relative to when the entry was originally authored and what their threshold for disclosure was, this would contribute to raising their COI standards to the level of most journals.

It did not escape notice that less than one-third of physician-authors were women. Women were the recipients of less than one-fifth (17.3%) of remuneration paid by industry to DynaMed contributors. Similarly, women received one-fortieth (2.5%) of the total payments from industry to UpToDate contributors. The analysis on gender revealed an interesting difference in disclosure status between men and women. However, there are multiple ways to interpret this. Perhaps, men physicians disclosed more because they were more often the beneficiaries of industry dollars^24,25,26,27^ and in a position requiring disclosure, or perhaps, the difference was owing to the underrepresentation of women physician-contributors. The last explanation has been supported in prior studies regardless of the source being a printed text^2,3^ or website.^17^ Women accounted for less than one-fifth of authors to Harrison’s *Principles of Internal Medicine*.^2^ These gender differences in point-of-care databases extend on past disparities in first and last authorship of journal publications, conference presentations, grant funding, time to promotion, and salary.^28,29^

### Recommendations

Our recommendations for evidence-based, point-of-care websites are 2-pronged. First, the disclosure information provided for each contributor could be made more robust by providing a verified date or timeframe for a nothing-to-disclose status, by hyperlinking to the OP or DFD pages for those who have entries, and by displaying a monetary range of financial payments from industry (eg, $5000-$10 000) for those who have entries. Such changes would offer greater transparency to the website user who consumes the information. Second, the current COI policy should be reviewed and updated annually, and a verified “no-COI” editorial team should be established to crosscheck physician-contributors at random against entries, similar to what was performed in this study. Such a policy might result in content contributors erring on the side of caution and disclosing more openly and completely. Additionally, although the dearth of women contributors was consistent with prior studies,^2,3^ further studies should examine whether this scarcity impacts how or what content is presented in these widely used^17^ point-of-care databases.

### Limitations

This study has some limitations. One argument against the findings of this study indicating any ethical lapse among physicians may be that when physicians contribute content to reputable online point-of-care websites, such as UpToDate and DynaMed, they might subsequently then be hired by the health care industry for their expertise and financially compensated, all within the same calendar year, although we believe the frequency of this occurring was low. Similarly, a physician-contributor may have received payment from the health care industry in one subject area but written and/or reviewed content on a completely unrelated topic for a point-of-care website; this individual may accurately have no COI and yet be considered discordant in this study. In both these plausible examples, no direct COI has occurred which could conceivably impact the database content. Importantly, OP and DFD provide specific dates for the payments reported to a physician, but no such timeline is provided by UpToDate or DynaMed on when the content was initially published. Each site does provide a last-revised date for the article but not when the contributor declared their disclosure status nor what aspects of an article were updated. To work within these confines, the research team opted to focus on disclosure status and existence of an OP or DFD entry rather than the timeline. We concur with a report by Chengappa et al^9^ that there is potential room for improvements in point-of-care database COI transparency.

Additional limitations of this study include that the sample size of contributors, although focusing on diseases and conditions of substantial importance and much larger than prior research,^7,8,17^ was limited to only US-based physicians. Our objective was not to ascertain the veracity of disclosures for all the content on either UpToDate (>7300 contributors)^18^ or DynaMed but only a defined subset based on the top 50 causes of death in the US. These findings are limited to 2 widely used point-of-care databases^21^ and may not generalize to other databases^20^ or non-English databases. Future research should be completed that systematically explores how the point-of-care article is written and whether this might have been impacted by the presence of industry reimbursements.

Additionally, our data are only as accurate as the financial reporting provided by OP and DFD. Given that a small subset of contributors (3.4%) was neither discordant nor concordant, this may be a concern worthy of further empirical attention.

## Conclusions

This cross-sectional study found that 179 contributors to point-of-care databases were the recipients of nearly $78 million from pharmaceutical companies and medical device manufacturers, and these payments were often not disclosed in association with contributed content. Research in this area of medical authorship and COI needs to continue, with a particular emphasis placed on online medical resources. The complexity of COI is vast and can go unnoticed by many readers who are using these medical resources for first-line treatment options and diagnoses of their patients. Thus, a better systematic approach is essential to identify and make transparent COI, for example, by providing an exact date when disclosures were last assessed by the website’s editorial team. Such actions would ultimately help to bolster trust among individuals within the medical field as well as in the resources available to them. Physicians, other health care professionals, and their patients should have maximum confidence knowing that the evidence-based medical information they receive is free from any outside influences.

## Notes

Supplement. **eTable 1.** Amounts Paid by Industry to Discordant UpToDate Physician-Contributors as Reported by the Center for Medicare & Medicaid Services Open Payments Database, 2013-2018 **eTable 2.** Amounts Paid by Industry to Discordant DynaMed Physician-Contributors as Reported by the Center for Medicare & Medicaid Services Open Payments Database, 2013-2018
