# Supplementary material for: Examination of Potential Industry Conflicts of Interest and Disclosures by Contributors to Online Medical Resource Databases
Source: JAMA Netw Open. 2022 Jul 5;5(7):e2220155. doi: 10.1001/jamanetworkopen.2022.20155 (PMC9257578; doi:10.1001/jamanetworkopen.2022.20155)
Supplement: Supplement. — eTable 1. Amounts Paid by Industry to Discordant UpToDate Physician-Contributors as Reported by the Center for Medicare & Medicaid Services Open Payments Database, 2013-2018 eTable 2. Amounts Paid by Industry to Discordant DynaMed Physician-Contributors as Reported by the Center for Medicare & Medicaid Services Open Payments Database, 2013-2018 [file jamanetwopen-e2220155-s001.pdf]

Supplemental Online Content

VanDeMark SH, Woloszyn MR, Christman LA, et al. Examination of potential industry conflicts of interest and disclosures by contributors to online medical resource databases. *JAMA Netw Open*. 2022;5(7):e2220155. doi:10.1001/jamanetworkopen.2022.20155

eTable 1. Amounts Paid by Industry to Discordant UpToDate Physician-Contributors as Reported by the Center for Medicare & Medicaid Services Open Payments Database, 2013-2018

eTable 2. Amounts Paid by Industry to Discordant DynaMed Physician-Contributors as Reported by the Center for Medicare & Medicaid Services Open Payments Database, 2013-2018

This supplemental material has been provided by the authors to give readers additional information about their work.

© 2022 VanDeMark SH et al. *JAMA Network Open.*

eTable 1. Amounts Paid by Industry to Discordant UpToDate Physician-Contributors as Reported by the Center for Medicare & Medicaid Services Open Payments Database, 2013-2018

Unique ID	Total Payments ($)	General Payments ($)	Research ($)	Associated Research ($)	Owner/Investment ($)
112	3,120,614	101,794	55,717	2,963,103	0
89	376,260	7,761	1,093	367,406	0
38	308,909	220	0	308,689	0
67	184,999	173,929	167	10,903	0
13	159,077	123,369	3,180	32,528	0
32	106,004	106,004	0	0	0
120	104,149	1,494	0	102,655	0
60	97,654	15,211	37,210	45,233	0
79	65,097	63,284	1,813	0	0
6	60,762	60,762	0	0	0
81	57,577	18,629	0	38,948	0
123	37,363	37,363	0	0	0
18	29,093	14,343	0	14,750	0
83	19,499	19,499	0	0	0
76	16,635	16,635	0	0	0
63	12,572	12,572	0	0	0
30	10,966	0	0	10,966	0
126	10,247	4,010	6,237	0	0
28	7,375	7,375	0	0	0
55	4,977	4,977	0	0	0
69	4,749	4,749	0	0	0
41	3,978	1,270	0	2,708	0
21	3,237	3,237	0	0	0
2	2,700	2,700	0	0	0
84	1,570	1,570	0	0	0
56	1,356	1,356	0	0	0
74	1,008	1,008	0	0	0
114	793	793	0	0	0
24	469	469	0	0	0
11	430	430	0	0	0
23	338	338	0	0	0
129	337	337	0	0	0
104	276	276	0	0	0
121	255	255	0	0	0
25	118	118	0	0	0
22	83	83	0	0	0
53	69	69	0	0	0
72	43	43	0	0	0

© 2022 VanDeMark SH et al. *JAMA Network Open.*

111	33	33	0	0	0
57	27	27	0	0	0
107	23	23	0	0	0
62	16	16	0	0	0
82	16	16	0	0	0
44	10	10	0	0	0

© 2022 VanDeMark SH et al. *JAMA Network Open.*

eTable 2. Amounts Paid by Industry to Discordant DynaMed Physician-Contributors as Reported by the Center for Medicare & Medicaid Services Open Payments Database, 2013-2018

Unique ID	Total Payments ($)	General Payments ($)	Research ($)	Associated Research ($)	Owner/Investment ($)
138	630,424	319	0	630,105	0
153	546,237	56,516	361,678	128,043	0
149	524,690	270,626	0	254,064	0
170	235,118	182	1,973	232,963	0
140	216,765	92,272	0	124,493	0
182	154,627	2,380	0	152,247	0
145	95,150	28,879	0	66,271	0
166	85,675	85,675	0	0	0
180	58,911	58,911	0	0	0
187	47,760	9,619	0	38,141	0
189	46,584	24,884	0	21,700	0
190	43,304	41,079	0	2,225	0
134	14,845	1,286	10,421	1,460	1,678
161	13,741	13,741	0	0	0
177	9,780	0	0	9,780	0
169	7,858	7,858	0	0	0
144	7,703	7,703	0	0	0
148	1,403	1,403	0	0	0
176	1,331	1,331	0	0	0
183	743	743	0	0	0
175	562	562	0	0	0
193	469	469	0	0	0
179	380	380	0	0	0
147	355	355	0	0	0
171	278	278	0	0	0
164	126	126	0	0	0
154	116	116	0	0	0
186	110	110	0	0	0
174	97	97	0	0	0
143	85	85	0	0	0
151	78	78	0	0	0
185	69	69	0	0	0
192	51	51	0	0	0
191	27	27	0	0	0
146	26	26	0	0	0

© 2022 VanDeMark SH et al. *JAMA Network Open.*
